# Nanomaterials: a review of emerging contaminants with potential health or environmental impact

**DOI:** 10.1186/s11671-023-03787-8

**Published:** 2023-04-21

**Authors:** Amer S. El-Kalliny, Mahmoud S. Abdel-Wahed, Adel A. El-Zahhar, Ibrahim A. Hamza, Tarek A. Gad-Allah

**Affiliations:** 1 Water Pollution Research Department, National Research Centre, 33 El Buhouth St., Dokki, Giza, 12622 Egypt; 2 Department of Chemistry, College of Science, King Khalid University, P.O. Box 9004, Abha, 61413 Saudi Arabia

**Keywords:** Nanomaterials, Emerging contaminants, Nanomaterials classification, Nanomaterials impact, Nanoparticles effect, Nanomaterials control

## Abstract

Nanotechnologies have been advantageous in many sectors and gaining much concern due to the unique physical, chemical and biological properties of nanomaterials (NMs). We have surveyed peer-reviewed publications related to “nanotechnology”, “NMs”, “NMs water treatment”, “NMs air treatment”, and “NMs environmental risk” in the last 23 years. We found that most of the research work is focused on developing novel applications for NMs and new products with peculiar features. In contrast, there are relatively few of publications concerning NMs as environmental contaminants relative to that for NMs applications. Thus, we devoted this review for NMs as emerging environmental contaminants. The definition and classification of NMs will be presented first to demonstrate the importance of unifying the NMs definition. The information provided here should facilitate the detection, control, and regulation of NMs contaminants in the environment. The high surface-area-to-volume ratio and the reactivity of NMs contaminants cause the prediction of the chemical properties and potential toxicities of NPs to be extremely difficult; therefore, we found that there are marked knowledge gaps in the fate, impact, toxicity, and risk of NMs. Consequently, developing and modifying extraction methods, detection tools, and characterization technologies are essential for complete risk assessment of NMs contaminants in the environment. This will help also in setting regulations and standards for releasing and handling NMs as there are no specific regulations. Finally, the integrated treatment technologies are necessary for the removal of NMs contaminants in water. Also, membrane technology is recommended for NMs remediation in air.

## Introduction

Nanotechnology is one of the most exciting and fast-moving areas of science today. It has been advantageous in many sectors, like medicine, the military, electronics, food, chemicals, energy, and a wide variety of other scientific fields [1–3]. However, nanomaterials (NMs) are considered emerging environmental contaminants.

The word “emerging” means newly formed or prominent. Hence, the term “emerging contaminant” or generally “emerging environmental contaminants” can be demonstrated as a chemical or a material characterized by a perceived, potential or real threat to human health or the environment or by a lack of published health standards [4–6]. The discovery of new contamination sources or new pathways for a contaminant identifies it as emerging. Also, developing a new detection method or treatment technology for a contaminant gives the same identification as an emerging contaminant. There is a long list of environmental emerging contaminants including, but not limited to, pesticides, pharmaceuticals, personal care products, plasticizers, fragrances, flame retardants, hormones, algal toxins, siloxanes, different trace elements such as radionuclides and rare earth elements [7–10]. Among these different types of emerging contaminants are nanoparticles (NPs) and NMs. NMs are defined according to the International Organization for Standardization (ISO) and the European Union (EU) as a “material with any external dimension in the nanoscale or having an internal structure or surface structure in the nanoscale” [11, 12]. Also, NPs are defined by ISO and EU as a “nano-object with all three external dimensions in the nanoscale”, where the nanoscale is the size range from approximately 100 nm [12, 13].

**Fig. 1:**
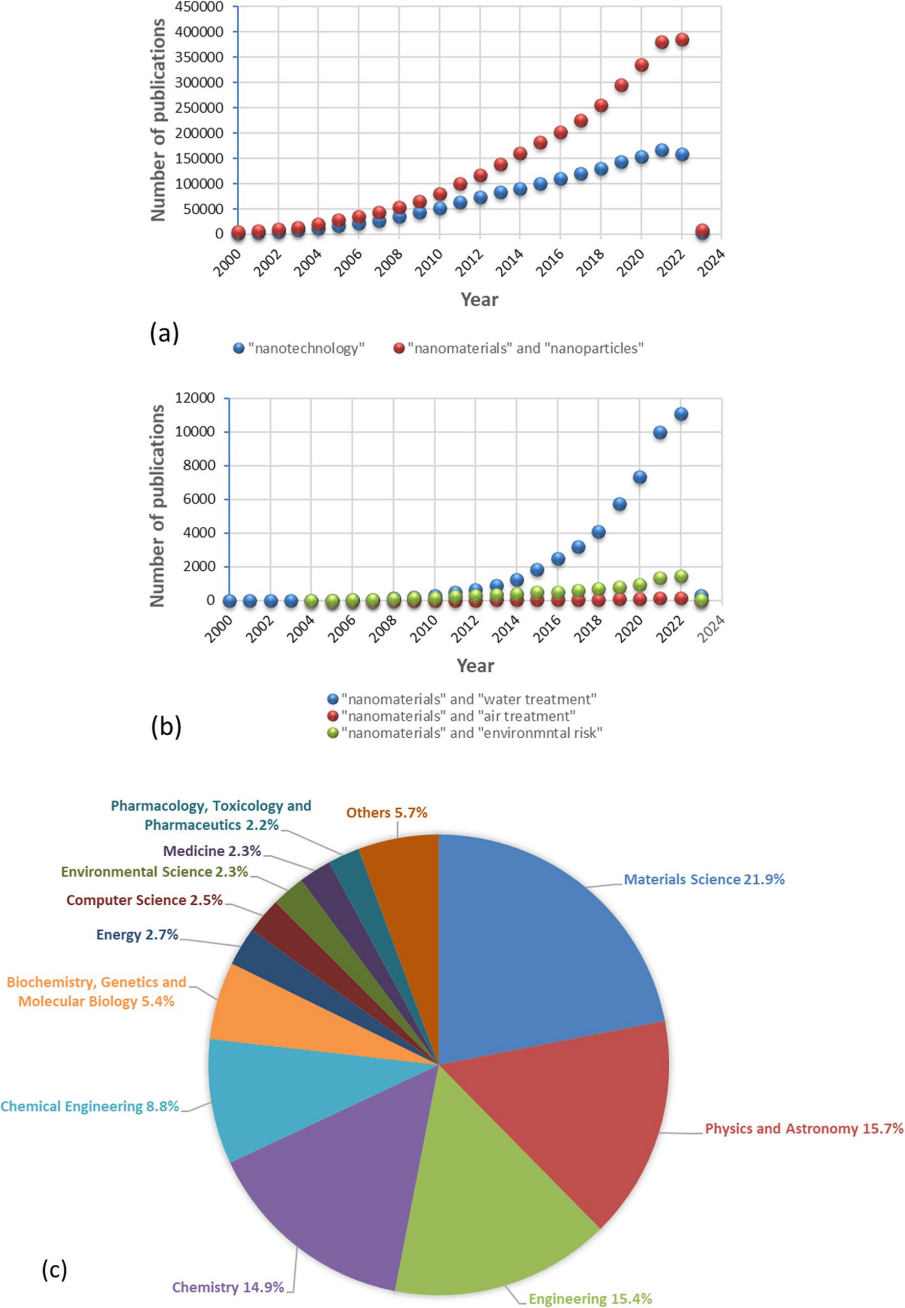
Comparison of the number of peer-reviewed publications. (Data analysis of publications has been done using the Scopus scholar search system with the terms: **a** “nanotechnology”,“NMs”, and “NPs” from 2000 to January 2023, **b** “NMs” and “water treatment”, “NMs” and “air treatment”, and “NMs” and “environmental risk” from 2005 to January 2023, **c** the percentage of documents per subject area for nanotechnology term

As we devote this review for NMs as emerging environmental contaminants topic, a survey has been done of peer-reviewed publications related to “nanotechnology”, “NMs and NPs”, “NMs and water treatment”, “NMs and air treatment”, and “NMs and environmental risk” in the last 23 years, as shown in Figs. 1a, b. There is an enormous increase in the number of publications in the field of nanotechnology since 2000 (Fig. 1a). This leads to an exponential increase in NMs- and NPs-related research publications in the past decade. This is because NMs are beneficial in many fields. Figure 1a shows that the highest number of publications used the “NMs and NPs” term, which indicates the importance of using this term for identifying the relevant articles. For example (and not limited to), NPs can be used in catalytic pollution prevention applications (e.g., metal oxides, clay materials, carbon materials, metal–organic frameworks, ultrafine noble metal, quantum dots, zero-valent metallic and bimetallic, etc.) [1, 14–17] and can also be used in energy and manufacturing-related applications [14, 18]. Consequently, this reflected an increase in the number of publications related to environmental risk (see Fig. 1b). There are a significant number of publications related to NMs, which can be used for water and air treatment (see Fig. 1b). This number of publications and studies is increasing. These studies have focused on different sides of NMs like their new applications as catalysts, adsorbents, disinfectants, and ion exchangers in air and water treatment technologies [19–22]. Figure 1c shows the percentage of documents per subject area for the nanotechnology term. The highest percentage of documents related to nanotechnology term is for materials science (21.9%). Comparable percentages of about 15% are for physics and astronomy, engineering, and chemistry which show the attempts for increasing the applicability of nanotechnology. While nanotechnology is a potential technique for environmental remediation, environmental science only accounts for 2.3% of all research. For this context, there are few numbers of publications concerning NMs as emerging contaminants relative to that for NMs applications. Most of the research work is focused on developing new applications for NMs and new products with peculiar features.

There are some published articles about NMs as emerging contaminants from different points of view. In the following, we present some examples of them. Sébastien Sauvé and Mélanie Desrosiers presented a review about how one defines emerging contaminants in general and pointed out briefly that NMs are emerging contaminants [7]. Buzea et al. [23] reported in a review an overview of NPs and their origin, activity, and biological toxicity. Vlachogianni and Valavanidis presented an overall review of the current knowledge associated with human health and toxicity risk of the engineered NPs [1]. Also, the risk assessment of NMs with regard to ecology, human health, and the environment was presented in [2, 22, 24]. Several underlining technical issues were discussed by comparing the properties and behavior of manufactured NPs with anthropogenically produced NPs in the air [25, 26]. An overview of emerging manufactured and biofuel NPs in the air was presented in [25, 26]. In [27], a review by Peralta-Videa et al. included the most recent publications for the biennium 2008–2010 reported on risk assessment, stability and characterization, toxicity, the fate of NMs in terrestrial ecosystems, and novel engineered nanomaterials (ENMs). The methods for the detection of NMs were reported in [26, 28]. Richardson and Ternes reported a series of review articles (2009–2017) for the analysis of emerging contaminants in water and surely NMs had a part in these reviews [29–33]. Jeevanandam et al. [34] have reviewed the classifications and the history of NMs and presented different sources of NPs and their toxic effects on mammalian cells and tissue.

The scientific community must pay attention to emerging contaminants in the environment. A number of important topics should be addressed, including detection and analysis techniques, treatment technologies, and risk assessments for emerging contaminants. As a result, this review article sheds light on NMs as emerging contaminants in the environment. In this paper, we describe the fate of NMs as emerging contaminants in the air, water, and soil. Also, the definition and the classification, and some examples of NMs usage will be presented. Furthermore, the routes of human exposure, ecotoxicity, health effects of NMs contaminants, and occupational health effects and safety issues will be highlighted. Finally, guidelines or regulations for NMs with the possible methods of detection and technologies for controlling NMs contaminants in the environment will be discussed.

## NMs

This section presents the characteristics, classification, and examples of NMs to provide a comprehensive understanding of the type of emerging contaminants being studied. It is crucial to standardize the terminology used for particle size across nanotechnology, health, and environmental sciences [2, 23]. The EU's definition of NMs is based on particle size, which can either be classified as NPs (smaller than 100 nm) or microparticles (MPs) (larger than 100 nm) [12]). NPs’ size is comparable to that of viruses, DNA, and proteins, while MPs’ size is comparable to cells, organelles, and larger physiological structures. The difference in size between NPs and MPs leads to unique features. The peculiar features of NMs are mainly due to the reduction of particle size to lower than 100 nm. These features include thermal stability, high strength, high conductivity, and low permeability. The reduction in particle size is responsible for increasing the surface area-to-volume ratio, which makes NMs more reactive than bulk forms of the same materials. Accordingly, it is challenging to anticipate the chemical characteristics and toxicities of NPs, even if the bulk materials they are derived from are well understood.

### Material-based classification

NMs including NPs can be organized into four main material-based categories as follows [34, 35]:

i. Carbon-based NMs, which contain mainly carbon such as carbon nanotubes (CNTs), carbon nanofibers (CNFs), carbon black, fullerenes (C60), graphene (Gr), and nanodiamonds [36];
ii. Inorganic-based NMs, which mainly include metal NPs (e.g., Au and Ag), metal oxide NPs (e.g., TiO_2_ and ZnO), and semiconductors (e.g., Si and ceramics);
iii. Organic-based NMs, which are made up of mostly organic materials (e.g., liposomes, polymeric NPs, micelles, and dendrimers);
iv. Composite-based NMs, which are multiphase NPs and nanostructured materials. The composites may be any combination of carbon-based, metal-based, or organic-based NMs with any form of metal, ceramic, or polymer bulk materials.

### Dimension-based classification

**Fig. 2:**
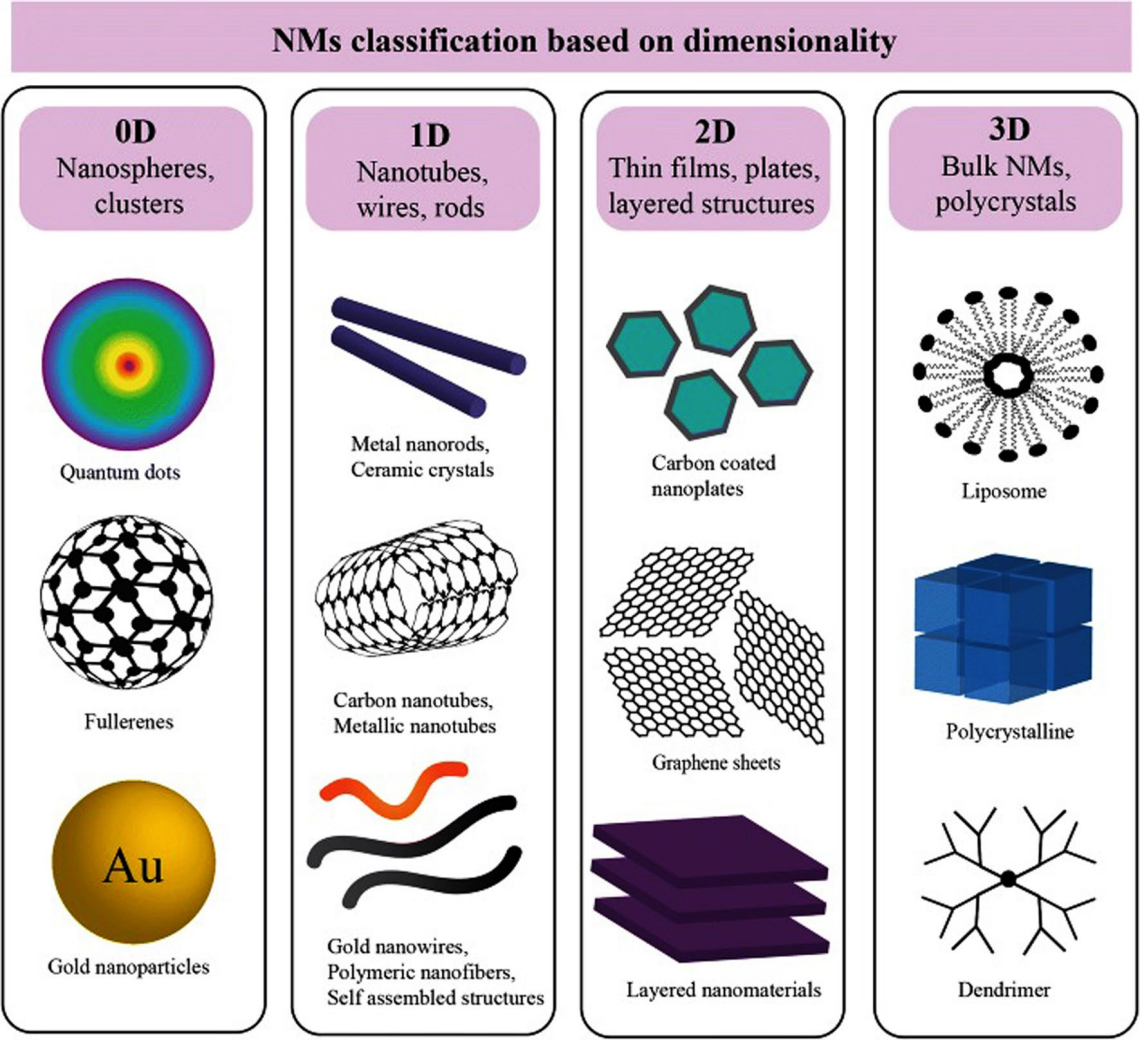
Classification of NMs according to their dimensions [51]

NMs can be classified according to their dimensions. For that purpose, Pokropivny and Skorokhod [2, 37, 38] reported the most comprehensive classification of NMs. As they stated, NMs can be classified as those materials with either zero-dimension (0D) in which all three dimensions are in a nanometric range such as quantum dots in light-emitting diodes (LEDs) [39], solar cells [18], and lasers [40]; one dimension (1D) in which two dimensions are in a nanometric range such as nanowires [41], nanorods [42], and nanotubes [43]; two dimensions (2D) in which one dimension is in a nanometric range such as nanothin films [44], nanosheets [45], and nanoplates [46]; or three dimensions (3D) in which all three dimensions are outside of a nanometric range, as shown in Fig. 2 [45, 47]. 3D NMs may consist of a group of nanofibers [48], nanotubes [49] or different distributions of NPs [50].

### Classification of NMs based on their source

In terms of their sources, NMs can be classified into the following three types [52].

I. Natural NMs [25] are naturally occurring materials on the nanoscale that are produced in nature either by biological species or through anthropogenic activities. They are produced in many natural processes such as photochemical reactions, volcanic eruptions, forest fires, and simple erosion, and by plants and animals, e.g., shed skin and hair [23].
II. Incidental NMs [25] are generated as side products or byproducts of anthropogenic processes [53, 54] and human activities such as trains, ships, aircraft, road vehicles, and even some natural processes such as forest fires [34].
III. ENMs [27] represent manufactured NMs that are deliberately produced with specific properties. They can be generated *by physical*, *chemical or hybrid* methods, such as milling (from their macro-scale counterparts) or self-assembly (from atoms and molecules). Besides, they may be discharged into the environment via either environmental and industrial applications or inappropriate handling of NMs [4]. Consumer products of various fields have been affected by ENMs such as cosmetics, textiles, food contact materials, improved diagnosis, and treatment of diseases. Also, ENMs are expected to be found with novel technologies, which are dedicated to waste remediation, the production of efficient energy storage, and advances in computer sciences [1].

The special characteristics of NMs make them potentially highly reactive in both environmental and biological systems which in turn alter the fate, the dispersion, and the toxicity of NMs, compared with their larger materials [55, 56]. Table 1 shows some examples of NMs with their physical and chemical properties and uses.

**Table 1:** Properties and common uses of NMs (source: [4])

Types of NMs (occurrence)	Example	Physical properties	Chemical properties	Uses
Carbon-based (natural or engineered)	Fullerenes/buckyballs (carbon 60, carbon 20, carbon 70); carbon nanotubes; nanodiamonds; nanowires	They exist as hollow spheres (buckyballs), ellipsoids, tubes (nanotubes); 1 nm nanowires or hexagonal structures (nanodiamonds). Excellent thermal and electrical conductivity	Carbon-based NMs are stable, have limited reactivity, are composed entirely of carbon, and are strong antioxidants	Biomedical applications, super-capacitors, sensors, and photovoltaics
Metal oxides (natural or engineered)	Titanium dioxide (TiO_2_); zinc oxide (ZnO); cerium oxide (CeO_2_)	Some have photocatalytic properties, and some have ultraviolet (UV) blocking ability. When used in sunscreen, nano-TiO_2_ and nano-ZnO appear transparent when applied on the skin	High reactivity; photolytic properties	Photocatalysts, pigments, drug release, medical diagnostics, UV blockers in sunscreen, diesel fuel additive, and remediation
Zero-valent metals (engineered)	Nanoscale zero-valent iron, emulsified zero-valent iron, and bimetallic nanoscale particles (BNPs). BNPs include elemental iron and a metal catalyst (such as gold, nickel, palladium or platinum)	Between 1 and 100 nm or greater, depending on the NM-type containing the zero-valent metal. Properties can be controlled by varying the reductant type and the reduction conditions	High surface reactivity. Popular starting materials used in production include ferric (Fe [III]) or ferrous (Fe [II]) salts with sodium borohydride	Remediation of waters, sediments, and soils to reduce contaminants such as nitrates, trichloroethene, and tetrachloroethene
Quantum dots (engineered)	Quantum dots made from cadmium selenide (CdSe), cadmium telluride (CdTe), and zinc selenide (ZnSe)	Size: 10‒50 nm. The reactive core controls the material’s optical properties. The larger the dot, the redder (lower energy) its fluorescence spectrum	Closely packed semiconductor whose excitons (bound electron–hole pairs) are confined in all three spatial dimensions. Possible metal structures include CdSe, CdTe, CdSeTe, ZnSe, InAs, or PbSe, for the core; CdS or ZnS for the shell	Medical imaging, photovoltaics, telecommunication, and sensors
Dendrimers (engineered)	Hyperbranched polymers, dendrigraft polymers, and dendrons	Size: 2‒20 nm. Highly branched polymers. Common shapes include cones, spheres, and disk-like structures	Highly branched; multifunctional polymers	Drug delivery, chemical sensors, modified electrodes, and DNA transferring agents
Composite NMs (engineered)	Made with two different NMs or NMs combined with nanosized clay. They can also be made with NMs combined with synthetic polymers or resins	Composite NMs have novel electrical, optical magnetic, mechanical, thermal, or imaging features	Multifunctional components; catalytic features	Potential applications in drug delivery, water purification and cancer detection. Also used in auto parts and packaging materials to enhance mechanical and flame-retardant properties
Nanosilver (engineered)	Forms include colloidal silver, spun silver, nanosilver powder, and polymeric silver	Size: 10‒200 nm Made up of many atoms of silver in the form of silver ions	High surface reactivity; strong antimicrobial properties	Medicine applications, water purification, and antimicrobial uses. They are used for a wide variety of commercial products

### Organisms and biological NPs-based classification

In living organisms, whether micro-sized (such as viruses, bacteria, and algae) or complex-structured (such as plants, insects, birds, animals, and humans), NPs and nanostructures are naturally formed [34, 38, 57]. Also, some living organisms are so small to be less than a few microns in size (e.g., viruses ranging from 10 to 400 nm and some bacteria from 30 to 700 μm) [23] and thus considered in the range of nanoscale and so-called nano-organisms. They include nanobacteria, viruses, fungi, algae, and yeast. New technologies and developments in instruments to visualize NMs help in identifying the morphology of these naturally formed nano-organisms [34].

## NMs impact on the environment

The fate and transport of NMs including NPs in the environment depend on particle size, surface chemistry, as well as abiotic and biological processes in the environmental matrix. Thus, NPs may exist in a form of suspension as individual particles or aggregate into larger sized NMs. It may also be existed in a dissolving state or react with natural materials [45, 58]. The tiny size of NPs causes slowness in their settling rate, and, consequently, they may suspend for a long time in water and air. Therefore, NPs can transfer easier and farther away than larger ones of the same material do [4].

NMs that exist in solid wastes and wastewater effluents are discharged directly or accidentally into the aquatic environment either by rainwater runoff or wind [59, 60]. The small size also affects the mobility of NMs through porous media and makes them able to strongly attach to and agglomerate with mineral surfaces [61]. For instance, the attachment of NMs to mineral surfaces slows down their mobility in groundwater aquifers [62]. This leads to the traveling of NMs to longer distances before becoming trapped in the soil matrix, and therefore soils with high clay content tend to stabilize NMs and allow greater dispersal [4].

### NMs in air

In urban atmospheres, diesel- and gasoline-fueled vehicles and stationary combustion sources contribute more than 36% of NPs. The natural NPs in the atmosphere are lower than that released from manufactured NPs. The health impacts of such NPs are still being investigated with regulatory concerns moving from traditional particulate matter (PM10) (˂ 10 µm) to PM5, PM2.5, and below, as the increased toxicity of the finer particles has been identified [59, 63].

The main source of atmospheric NPs is automobile exhaust. Diesel engines release NPs of 20–130 nm whereas gasoline engines release NPs of 20–60 nm. It has been found that during diesel and gas combustion processes, CNTs and fibers are released as byproducts. More than 90% of carbon NPs present in the atmosphere are diesel-generated particles, thus pollution from vehicles is a major cause of nanoparticulate contamination in the urban atmosphere [34]. In addition to the exhausts, cigarette smoking and building demolition produce anthropogenic NPs. There are about 100,000 chemical compounds in the form of NPs in the particle size range of 10–700 nm [64]. NPs and microparticulates (˂ 10 µm) are released by the demolishing of a large building [65]. The released NPs around the site of building demolition may contain lead, glass, respirable asbestos fibers and other toxic particles from household materials [65].

Airborne ENMs in indoor air and ambient atmosphere is occurring from the production and application of ENMs. There is no specific study on the transport and fate of airborne ENMs in the ambient atmosphere [66]. However, Kalavrouziotis found that the catalytic converters, used for minimizing the emitted fumes by the car exhaust, have an emission for the platinum group elements. Due to this, these platinum group elements are emitted in the form of particulate matter and accumulate in the soil, plants, and air [67]. This particulate matter is being transported over long distances and has increased significantly especially along the roadside of highways during the last ten to fifteen years as has been detected recently. Besides, ENMs may form aggregates with dust particles [68, 69] and behave like an aerosol in an ambient atmosphere. Also, the fate and transport of aerosol have been studied in a review article by Buzea et al. [23], which may be used to understand the behavior of airborne ENMs.

### NMs in soil

In soils, natural NPs such as clays, organic matter, iron oxides, and other minerals play an important role in biogeochemical processes. Colloids in soil may enhance the movement of contaminants in soils and other porous media. The sorbed contaminants into colloids can be moved when conditions for colloidal transport are favorable [59, 70, 71].

Soil is considered a sink for ENMs and a possible source of groundwater contamination with such materials. Thus, it is essential to understand the transport behavior of ENMs in soil and connect that with the potential impact on the food chain and groundwater [66]. Tourinho et al. [61] presented from the literature the fate and effects of metal-based NPs (e.g., silver, zinc oxide, titanium dioxide, iron oxide) in soil. Also, surface chemistry plays an important role in the mobility of NMs in porous media. In water, for example, the coating of NMs with TiO_2_ could be problematic, but not in soil [72].

### NMs in water

In aquatic systems, a colloid has particles ranging from 1 nm to 1 µm. Aquatic colloids include naturally occurring materials (i.e., proteins, humic acids, fulvic acids, and peptides), and inorganic materials in colloidal form (i.e., manganese oxides and hydrous iron). These materials are important binding phases for both organic and inorganic contaminants due to their small size and large surface area per unit mass [59, 73].

In freshwater, the NPs aggregates deposited to the bottom and accumulated slowly in the sediment; this affected negatively the benthic species. While, in the marine ecosystem, NPs will possibly accumulate in between cold and warm currents [74]. NPs in marine ecosystems could be also recycled through biota [59]. Thus, this can increase the risk of the species in the interface between cold and warm currents such as tuna [74]. Nam et al. [75] discovered that a high level of TiO_2_ NPs was present in the sediment layer due to the settling of NPs in a simplified microcosm system. This system was designed to “assess the bioaccumulation of TiO_2_ NPs in multiple model species”. Also, Nam et al. [75] show that engineered NPs such as TiO_2_ NPs and Ag NPs can also travel through the feeding patterns of aquatic organisms. This study also showed that algal cells concentrate NPs due to the adhesion of NPs to the cell wall. Thus, the stability of ENMs in aqueous environments plays a role in their fate and transport in aqueous environments. The ENMs of large aggregates will be deposited quickly, and their transport and bioavailability will be greatly restricted [66]. In contrast, well-dispersed and small aggregates of ENMs will be widely transported and have higher chances to interact with and cause more risk to organisms. Few studies have been done on the possible existing forms of ENMs in the real natural water system [66]. However, aggregation is a common process for ENMs in water and causes a reduction of overall surface area and thus will limit the reactivity of such materials [66]. The influencing factors on the fate and transport of ENMs in an aqueous environment such as natural organic matter and pH were emphasized in an article review reported by Lin et al. [66].

The effects of NMs on wildlife species are still being conducted by research. Some research studies have reported a harmful effect on aquatic species, “trout” in particular, after being exposed to TiO_2_ NPs [76]. The biodegradability of NMs is still under research investigation; for instance, some biodegradable fullerenes (e.g., C60 and C70) have been found to take several months to decompose, while metals and metal oxides are not biodegradable [4]. Zero-valent iron NPs are used for on-site remediation, yet little is known about their fate and transfer in the environment [4]. Also, simulating models can help in studying the fate of NMs in the environment. Recently, Avant et al. (2019) [77] applied the Water Quality Analysis Simulation Program 8 (WASP8) for simulating exposure concentrations of carbon-based NMs in surface waters and sediments. They studied the fate and transport of multiwalled carbon nanotubes (MWCNT), graphene oxide (GO), and reduced graphene oxide (rGO) in four aquatic ecosystems in the southeastern United States. They found that MWCNT existed predominantly in the sediments of the river and seepage lake. They estimated that the recovery periods would be 37 years for lakes and 1‒4 years for rivers to reduce sediment NM concentrations by 50% suggesting that carbon-based NMs have the potential for long-term ecological effects. Exposure to NMs is currently being explored and these may enter the air, water, and soil media from different routes. Thus, the routes of human exposure to such materials will be described in the following section.

## The interaction of NPs with biosystem

NPs can be located inside the cell in many locations such as cell membrane, cytoplasm, lipid vesicles, or within the nucleus, and hence can damage DNA or cause cell death [23, 78, 79]. The contact between NPs and cell membranes is a vital step before cellular uptake. The mechanism of cell uptake of NPs is assumed as adhesive interaction by Van der Waals forces, steric interactions, electrostatic charges, or interfacial tension effects [80]. Scientific Committee on Emerging and Newly Identified Health Risks, 2006, reported the effects of NPs properties on the interaction with living organisms, and a summary of these effects is presented below [80].

### The effect of NPs surface

The surface characteristics of NPs play a crucial role in NP-cell interactions and solubility. Altering the surface coating of NPs might affect their cytotoxic characteristics by affecting pharmacokinetics, diffusion, accumulation, and toxicity [81]. Colloidal behavior occurs as a consequence of changes in NP shape and size in the surface charge of the organism. It determines the response of the organism to NP shape and size in the form of cellular accumulation. It has been shown that surface chemistry affects NPs' absorption, colloidal behavior, plasma protein binding, and their ability to cross the blood–brain barrier. Also, a higher surface charge increases the cytotoxicity of NPs due to greater endocytic uptake [82]. There is therefore some evidence to suggest that positively charged NPs tend to accumulate more in tumors than negatively charged NPs, possibly as positively charged density is more easily separated in the interstitial space and, as a result, taken by tumor cells [83]. Also, due to the presence of negatively charged proteins on the outer membrane of gastrointestinal epithelial cells, positively charged NPs are more permeable to the gastrointestinal mucous barrier than neutral and negatively charged NPs [84]. Another significant variable that has an impact on pharmacokinetics and biodistribution is hydrophobicity. Plasma proteins tend to be absorbed by NPs that have two or more hydrophobic surfaces, resulting in a shorter bloodstream retention time [85].

### The effects of size and shape of NPs

Size and shape of NPs affect biodistribution, kinetics of release, and cellular uptake of NPs. Generally, NPs are brought into cells through the three most significant mechanisms: phagocytosis, diffusion, and fluid phase endocytosis.

Because of their small size, NPs are frequently misidentified as foreign agents by macrophages; however, MPs easily are taken up by reticuloendothelial systems [86]. The surface area of the particles increases as their size decreases, and a larger surface area facilitates particle diffusion into cells. For instance, a study conducted by Donkor and Tang [87] found that 30 nm single-walled carbon nanotubes (SWNT) were more likely to be internalized by cells and nuclei than 50 nm SWNT. Previous studies have shown that, when the diameter increases to 500 nm, caveolae-mediated processes (endocytosis) become dominant in the cellular internalization of microspheres coated with clathrin [88]. Additionally, NPs of 50 nm diameter target and act on human mesenchymal stem cells without endocytosis [89]. Although NPs larger than 1 m are difficult to directly enter cells, they can interact with proteins that are taken up by the cells. NPs larger than 6 nm are incapable of being eliminated by the body leading to their accumulation in some organs [90]. Studies on the bio-distribution and bioaccumulation of AuNP of various sizes in the blood were conducted showing that smaller ones accumulated more significantly in all organs and stayed longer in the blood circulation [91]. Similarly, regardless of the sort of functional groups on the particle surface, cellular uptake of NPs increases in cancer cells as they become smaller [92]. For instance, the capacity of a cell to uptake a carbon nanotube depends on its length. Compared to longer ones, submicron multiwall carbon nanotubes have demonstrated more effective cell penetration [93].

On the other hand, NPs can be found in a variety of forms as mentioned before, this might also have an impact on how they are eliminated, internalized, and endocytosed. For instance, it has been proposed that spherical NPs internalize more quickly and easily by endocytosis than rod-shaped NPs, which is consistent with the longer membrane wrapping time needed for the elongated particles. Compared to spherical NPs, the elongated NPs are more effective at adhering to the cells, this is because only a small portion of the binding sites on spherical particles may bind with target cell receptors due to their curved form. While the surface area of the longer NPs facilitates their multivalent interaction with the cell surface [94]. Sharp-shaped NPs can pass through endosome membranes and enter the cytoplasm. As a result, their exocytosis was relatively lower than that of spherical particles. Whereas, ellipsoidal NPs have lower cell absorption than spherical NPs [95].

### Effect of medium/corona

When NPs reach a biological fluid like serum, their surface soon becomes covered with a coating of biomolecules such as proteins and lipids, with different affinity interactions [96]. These biomolecules, known as bio-corona, have been shown to have a significant influence on the biological activities of NPs, including complement activation, bio-distribution, contact with cell surface receptors, and cellular absorption of NPs [97]. NP physicochemical characteristics can influence the kind of corona. The production of the biomolecular corona is related to the size and surface modification of NPs. Further, biomolecule adsorption might cause NPs to grow in size, altering their pharmacokinetic and therapeutic efficacy in vivo.

Depending on how long the protein exchanges last, the protein corona is divided into hard and soft categories. A layer of proteins with strong affinity and a slow rate of exchange is called the hard corona. As the layer that is closest to the NP surface, the hard corona proteins are particularly prone to irreversible, thermodynamically advantageous conformational changes based on the chemistry of functionalization, the hydrophobic or hydrophilic properties of the proximal biological fluid, and the temperature [98]. The soft corona is a low affinity layer of proteins that exchange rapidly over time. Soft corona is indirectly connected to the NP via a certain (low) degree of biomolecule interactions.

Depending on the mode of administration, NPs are exposed to interactions with various biomolecules. As a result, protein concentration, particle size, NM type, and surface properties all have a role in defining biomolecule layers and protein corona density [99]. Another important aspect influencing protein corona formation is the biological environment, which includes media components, temperature, pH, and the physiological state of the medium. Therefore, understanding the link between many features of NMs and a specific biological environment is critical for understanding their stability, persistence, and behavior in the biological environment [98].

The development of NP protein corona complex can impact cellular absorption of NPs. The cellular absorption of NPs can be hindered by changes in the structure of adsorbed proteins. Furthermore, the unfolding of the adsorbed protein corona may influence NP accessibility to surface receptors. Moreover, the cellular attachment of NP protein complexes is a non-specific process that is dependent on the amount of adsorbed proteins rather than the kind of protein on the surface of the NPs [100].

### Solubility and persistence of NPs

There is a considerable solubility for many NPs and, therefore, their interaction with the living system is close to that with bulk chemical agents. Thus, the toxicological testing procedures can be well applied to that type of NPs. The biological effects of biodegradable NPs are influenced by NP structure and degradation byproducts. NPs with very low solubility or degradability, in contrast, could accumulate within biological systems and remain there for extended periods of time. These types of NPs must have the greatest concern [80, 101]. NPs are cleared from the human body via the renal and hepatobiliary transport system, which need to be completed in an appropriate time for clinical approval. The drug conjugated NPs therefore have to be engineered to avoid rapid elimination and prolonged body maintenance.

## Ways of human exposure to NPs

**Fig. 3:**
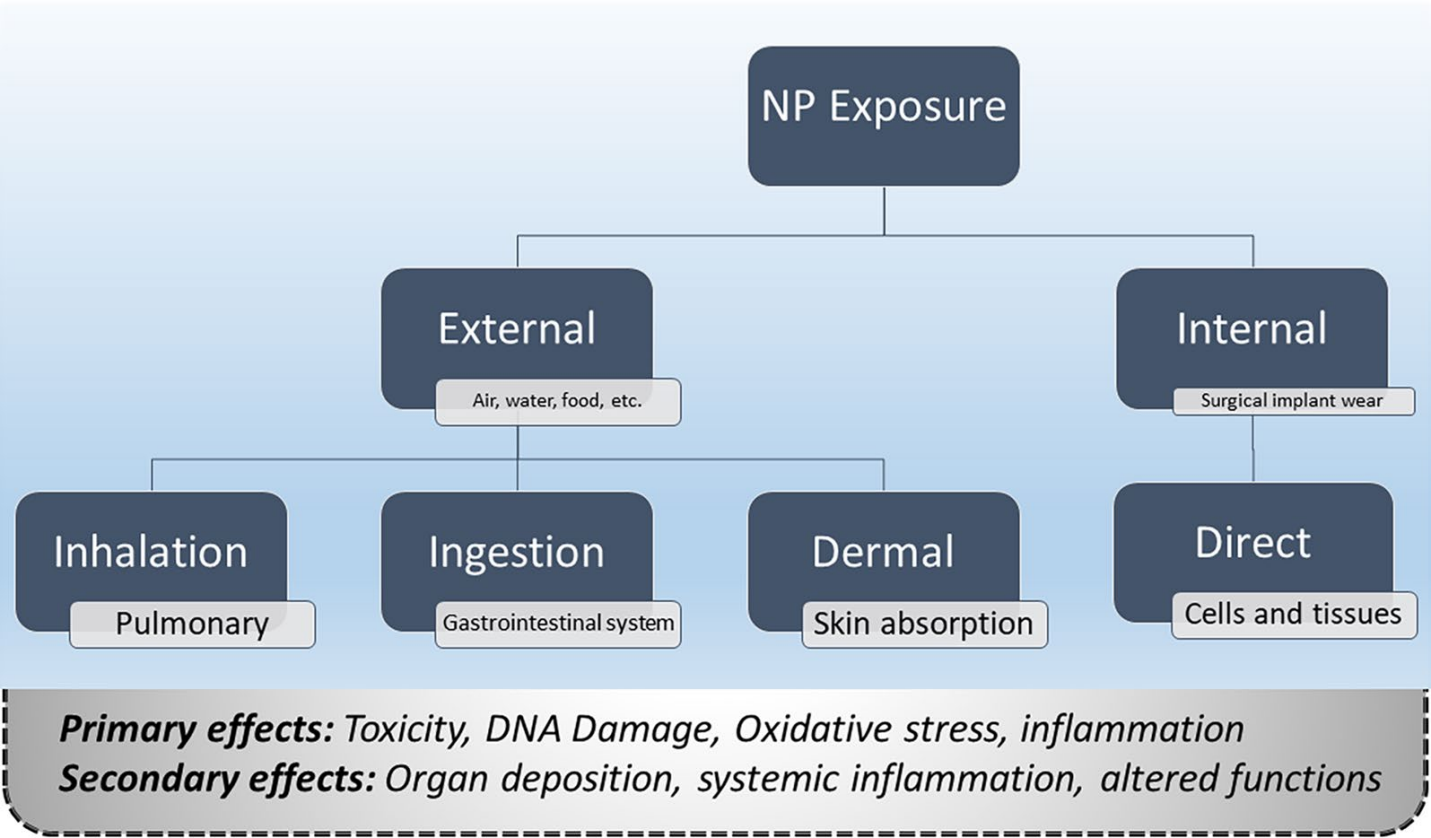
Routes and potential detrimental effects of NP exposure

Human exposure to NMs depends on the source and activities of the person. It may occur through inhalation, skin absorption, ingestion or injection. The most widely recognized route of human exposure is inhalation at the workplace [4]. Due to their small particle size, NMs can pass through both the blood–brain barrier (BBB) and the placenta. Liu et al. showed that TiO_2_ NPs may pass the BBB of mice when injected with high doses [102, 103]. Figure 3 shows two categories, primary and secondary, for the effects of NP exposure [104]. This categorization depends on the extent of exposure. Primary effects come from direct cellular NP contact. They may include toxicity, oxidative stress, DNA damage, and inflammation. Secondary NP exposure results from the translocation of NPs through tissue barriers into the blood, where they can circulate and eventually deposit in other organs. They may include toxicity at the site of NP deposition, in organs such as the liver, spleen or kidneys [104].

Inhalation, ingestion, and dermal exposure routes occur through external NP sources, while internal exposure can happen when orthopedic or surgical implant wear NPs are released locally from the implant site [104]. Recently, CNTs of anthropogenic origin were found in the bronchoalveolar lavage fluids of asthmatic Parisian children. The results suggested that there is routine human exposure to CNTs [34]. Azarmi et al. [53] studied the exposure levels of operatives on-site and the dispersion of ultrafine particles (˂ 100 nm) into the surrounding environment of building activities during the mixing of fresh concrete and the subsequent drilling and cutting of hardened concrete. This research article is considered as a step toward establishing number and mass emission inventories for particle exposure during construction activities.

Animal and human studies suggest that alveolar translocation of NPs leads to circulatory access. Then, NPs distribute throughout the body, including the vasculature, heart and they may reach the liver and spleen, the two major organs for detoxification, and bone marrow. However, the extent of extrapulmonary translocation mainly depends on particle size, surface characteristics, and chemical composition of NPs [105]. The inhaled carbon NMs by humans remain in the lung, less than 1 percent of the inhaled dose may reach the circulatory system [106].

**Fig. 4:**
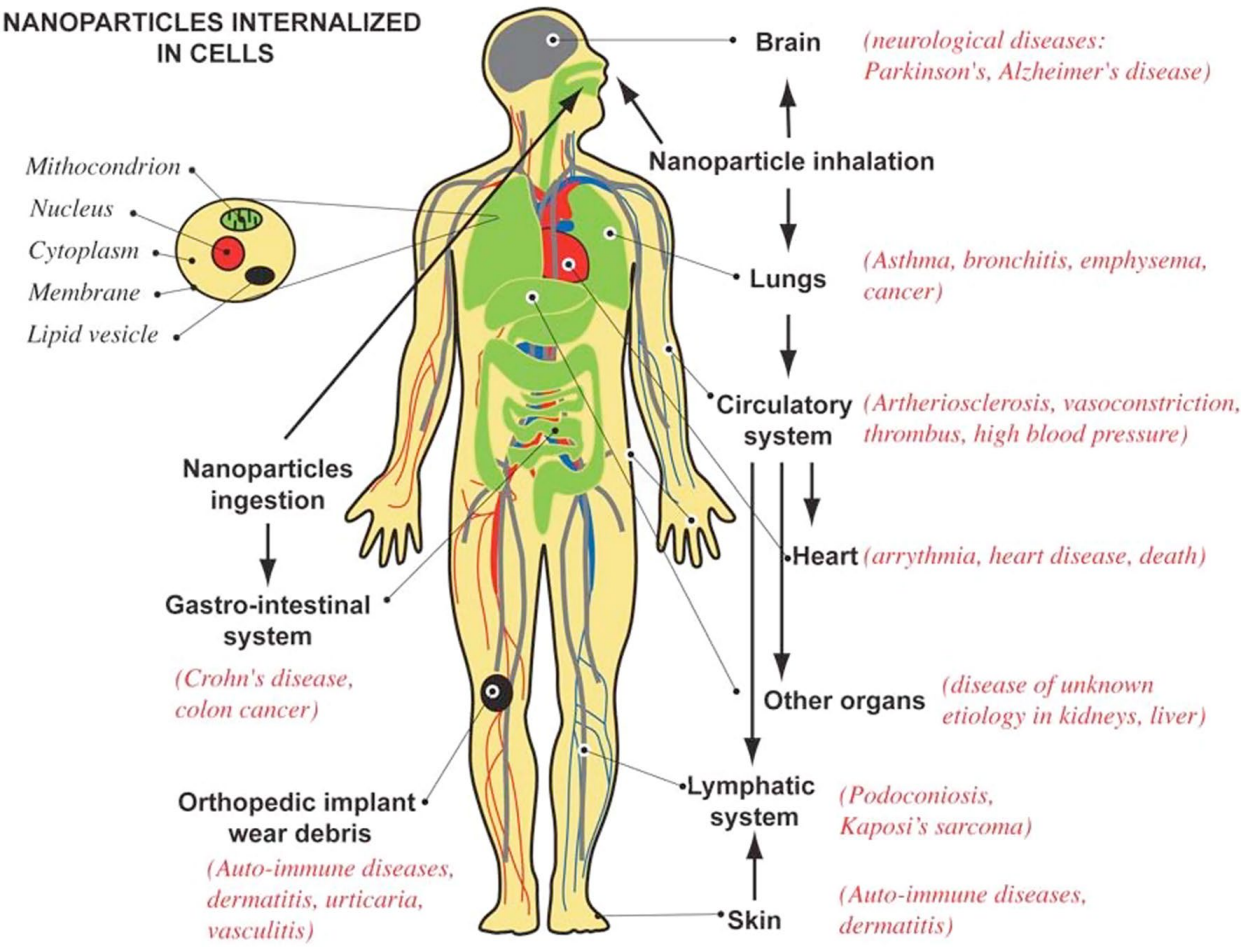
Schematics of the human body with pathways of exposure to NPs, affected organs, and associated diseases from epidemiological, in vivo and in vitro studies. *Reprinted with permission from* [23]*. Copyright [2007], American Vacuum Society."*

Dermal exposure to NMs can happen by using the products of sunscreen (e.g., TiO_2_ and ZnO). This level of exposure depends on the condition of the skin and the characteristics of the sunscreen. NM migration to the dermis may be prevented in the case of healthy skin. In contrast, NMs can penetrate the dermis and access regional lymph nodes in the case of damaged skin, as suggested by quantum dots and nanosilver [105, 107]. Ingestion exposure may also occur from consuming NMs contained in drinking water or food (e.g., fish) [62]. The pathways of exposure to NMs and a summary of the possible adverse health effects associated with inhalation, ingestion, and contact with NPs were presented in Fig. 4 [23]. Also, the entry mechanisms of inorganic NPs in the human body, such as TiO_2_ NPs, SiO_2_ NPs, ZnO NPs, Ag NPs, Au NPs, and quantum dots NPs, have been reviewed by De Matteis [108]. In this context, there is a broad range of applications for TiO_2_NPs and ZnO NPs in commercial products such as sunscreens, food additives, and paints. In addition to food and water disinfectants, Ag NPs are commonly used in the textile industries, diagnostic biosensors, imaging probes, and conductive inks. Therefore, they can enter the human body through inhalation, ingestion, and skin penetration. The majority of AuNP applications are in the medical field; photothermal therapy, bioimaging, and drug delivery are some of the most important ones. SiO_2_ NPs are found in food, powders, and healthcare items such as toothpastes, detergents, and cosmetics [109].

## Ecotoxicity of NMs

Little is known about the physiological responses to NPs. However, some conventional ecotoxicity and toxicity tests are useful for evaluating the hazards of NPs [80, 103]. For instance, Oberdörster [109] reported the toxicity of ENMs (e.g., fullerenes, C60). It was found that the LC50 (for 48 h) in *Daphnia magna* for fullerenes C60 is 800 µg/L. Also, Yamakoshi et al. [110] presented the bactericidal properties of fullerenes. There are a considerable large number of human toxicology studies as presented below. These studies demonstrate the uptake and effects of NPs at a cellular level, which can hold for species other than humans. However, the existing test methods for toxicity need modification and development [80].

Ecotoxicological effects of NMs have attracted attention since the pioneer study performed by Oberdörster [109]. In 2010, Stone et al. did a comprehensive nano-ecotoxicity survey covering 89 studies conducted between 2004 and 2009. After 12 years in 2021, the number of publications increased 69 times [111] indicating the importance of this topic. Therefore, the organization for economic co-operation and development (OECD) has recently released a guidance document for the toxicological testing of NMs in an aqueous environment and sediment [112].

Many ecotoxicological studies have not only followed the test guides (TGs) provided by OECD but also tried to improve those TGs. For instance, as an attempt to overcome the agglomeration phenomenon when manufactured NMs are mixed with algae in a culture medium for ecotoxicity tests, a dispersion method has been developed in terms of the type of dispersant, sonication time, and stirring speed [113]. The developed method was applied for 14 types of manufactured NMs specified by the OECD, namely aluminum oxide (Al_2_O_3_), carbon black, single-walled carbon nanotubes (SWCNTs), multiwalled carbon nanotubes (MWCNTs), cerium oxide (CeO_2_), dendrimers, fullerene, gold (Au), iron (Fe), nanoclays, silver (Ag), silicon dioxide (SiO_2_), titanium dioxide (TiO_2_), and zinc oxide (ZnO). The toxicity was measured through cell counts using *Raphidocelis subcapitata*. The half-maximal effective concentrations (EC50) were 18.0 ± 4.6 mg/L for SWCNTs and 316.6 ± 64.7 mg/L for TiO_2_. In addition, Pengiran et al. [114] have evaluated the acute toxicity of kenaf cellulose nanofiber (CNF) against *Daphnia magna* and *Danio rerio* following OECD Test No. 202: *Daphnia sp.* acute immobilization test. The EC_50 _and LC_50_ for *Daphnia magna* and *Danio rerio* were above 100 mg/L and classified as nonhazardous to the aquatic environment according to the Globally Harmonized System (GHS).

Silver NMs (Ag NM) have been engaged intensively in consumer products as antibacterial agents, which results in their occurrence in sewage sludge, and subsequently accumulated in the agricultural soils when sewage sludge is implemented as fertilizer. Accordingly, the toxicity of Ag NM in soil was assessed against the inhibition of ammonium-oxidizing bacteria (AOB) [115]. However, not much toxicity was observed for Ag NM. The aquatic ecotoxicity of manufactured silica NMs and their interactions with organic pollutants has been also studied in different studies [116–118]. In their studies, they investigated the ecotoxicity of nine silica NMs with different size, charge, surface modification, and shape in experiments with bacteria (*Pseudomonas putida*), algae (*Raphidocelis subcapitata*), crustacean (*Daphnia magna*), and fish gill cells (*Oncorhynchus mykiss*). The output of their studies is summarized in Table 2. As indicated in this table, the toxicity is dependent on the surface area, surface chemistry, and exposed organism/cell type.

**Table 2:** EC20 and EC50 values of silica NMs after exposure to bacteria, algae, and fish cells. Concentrations are presented in mg/L

Test organism	Silica NM						
	20 nm-aluminized	17 nm	18 nm-silanized	21 nm	30 nm	66 nm	88 nm
*Bacteria*							
EC20	> 500	> 500	> 500	> 500	> 500	> 500	> 500
*Algae*							
EC20	> 500	295	> 500	> 500	> 500	> 500	> 500
EC50	> 500	> 500	> 500	> 500	> 500	> 500	> 500
*Daphnia*							
EC50	> 10 000	> 10 000	> 10 000	> 10 000	> 10 000	> 10 000	> 10 000
*Fish cells*							
EC20	5	5	> 100	6	6	31	35
EC50	15	13	> 100	16	21	92	91

Unfortunately, the scattered data in the literature makes it difficult to have clear information about the ecotoxicity of NMs. This is because NMs have a wide range of properties affecting their interaction with the tested organisms.

## The health effects of NMs

The scientific data for NMs that may present adverse health effects to humans under realistic exposure scenarios are not sufficient. However, NMs can induce different levels of cell injury and oxidative stress depending on their charge and particle size. Also, surface excitation by UV light can modify the surface properties, aggregation, and biological effects of NMs [103, 105, 119]. NMs can form reactive oxygen species (ROS), which can lead to cell membrane damage upon contact. The lipid peroxidation process is happened by ROS, which can oxidize double bonds on fatty acid tails of membrane phospholipids. This process increases the permeability of the membrane, making cells more susceptible to osmotic stress or hindering nutrient uptake. It also activates reactions that generate other free radicals, leading to more cell membrane and DNA damage [59]. Moreover, the uptake-and-damage increases when cultured cells are exposed to NMs of various metals (e.g., Fe, Mn, and Co) with TiO_2_ containing SiO_2_ NPs and the corresponding pure oxides. Experiments elucidated the role of NPs as carriers for heavy metal uptake. NMs may be absorbed and gain access to tissues that metals alone cannot normally reach in an uptake-and-damage mechanism called the “Trojan Horse effect” [120]. Also, the accumulation of ROS in tissue was demonstrated by Mao et al. [121] A variety of ROS-mediated stress responses could be caused by Ag NPs, including apoptosis, DNA damage, and autophagy.

Quantum dots NPs are made of cadmium or lead, which are well-known toxins. The protective coatings of quantum dots can degrade in light and oxidative conditions, releasing these toxic metals into cells and organisms and causing toxic effects [122]. However, estimates of the releases of these metals from NMs are very crude [59]. In the case of immunotoxicity, research has shown that NPs can stimulate and/or suppress immune responses by binding to proteins in the blood [123].

Some of the health problems were observed in humans due to high exposure to exhaust, where automobile exhaust is major in densely-populated cities, such as cardiopulmonary mortality; childhood cancers due to prenatal and postnatal exposure to exhaust; myocardial infarction; and proinflammatory, prothrombotic, and hemolytic responses [34]. The relation between these health problems and NPs is demonstrated by concentration measurements of NPs near highways, where the concentration decreases exponentially over several hundred meters from the traffic [124]. Besides, the CNTs of anthropogenic origin can cause granulomatous reactions, oxidative stress, and inflammation, leading to fibroplasia and neoplasia in the lungs [125]. Also, a polynuclear aromatic (benzo[a]pyrene) exists in diesel exhaust, which makes it more toxic than those gas engine exhausts [126].

Cigarette smoke, containing NPs, can lead to chronic respiratory illness, cardiovascular disease, pancreatic cancer, genetic alterations, middle ear disease, and exacerbated asthma [34].

On the other hand, biological NPs, or nanobacteria, are pervasive within organisms, animals, and humans as they are identified in serum, blood, and organs. They are suspected to cause calcifications diseases such as artery plaque, heart valves, aortic aneurysms, chronic prostatitis, renal stone formation, ovarian, and breast tumors [23]. There are evidence that nanobacteria may act as nucleation sites for stone formation or plaque, while the exact mechanisms relating nanobacteria to these above diseases are unknown [127]. Also, diatoms have a health risk to workers in diatomaceous earth mining and processing [128]. Also, biogenic magnetite is related to neurodegenerative diseases [129].

### Occupational health and safety issues

The most extensive exposure to hazards is most likely to occur in the working environment. Therefore, it is expected that workers in nanotechnology-related industries and small workshops are intensively exposed to ENMs. Workers in nanotechnology-related industries and small workshops are exposed to ENMs. These NMs are characterized by novel sizes, shapes, and physical and chemical properties. Therefore, occupational toxicologists are the first to discover adverse effects on the occupational health and safety of ENMs [1, 130]. During the processes of manufacturing, NPs can penetrate the respiratory system and via the blood can move into other organs. Also, other indications were shown by studies that NPs can penetrate through the skin [1]. The ENMs (e.g., MWCNTs) have a risk involved by pulmonary exposure. Abdominal mesothelioma was observed in mice by MWCNTs exposure and was typically recorded for workers exposed to asbestos in the past [131].

There are many actions to minimize NPs’ risk for workers. For example, personal protective equipment and engineering controls are recommended by the National Institute of Occupational Safety and Health (NIOSH), USA, to significantly decrease workplace exposure to CNFs and CNTs. According to the available information on health risks, developing prevention strategies seem to be crucial to minimize NMs exposure in the workplace such as exhaust ventilation, enclosure, and respirators, as well as worker training for good handling practices. In the UK, the safe use and handling of manufactured NMs are controlled by a framework for occupational health and safety in the workplaces done by the Health and Safety Executive (HSE) [1].

## Guidelines or regulations for NMs

NMs are accompanied by many characteristics such as high chemical and biological reactivity. Also, they have cellular, tissue, and organ penetration ability. These features make them environmental contaminants. However, there is no international regulation for NMs environmental contaminants. There are also no specific federal standards for NMs’ size [132]. However, some federal statutes apply to NMs depending on the specific media of application or release [4]. Table 3 presents some federal standards and guidelines for NMs [6].

**Table 3:** Some federal standards and guidelines for NMs [4, 6]

Organization	Material under guidelines	The focusing subject of guidelines	Remarks
The U.S. Food and Drug Administration (FDA)	Evaluation and use of NMs in FDA-regulated products	Focus on assessing safety, effectiveness, and quality of products containing NMs	There is no categorical judgment on the safety or hazards of NMs
United States Environmental Protection Agency (EPA)	CNTs	Subject to reporting in Section "Ways of human exposure to NPs" of Toxic Substances Control Act (TSCA)	
EPA	NMs	Safe Drinking Water Act	No maximum contaminant levels have been established for NMs
Federal Food, Drug and Cosmetic Act (FFDCA)	NMs that are used as pesticides	If their use as a pesticide will result in residues in food or animal feed, a tolerance (maximum residue level) must be established	
The California Department of Toxic Substances Control (CA DTSC)	CNTs, nanometal oxides, nanometals, and quantum dots	Requesting information related to chemical and physical properties, including analytical test methods and other relevant information	Issued formal request letters to the manufacturers of certain NMs
The Occupational Health and Safety Administration (OSHA)		Adopt federal safety standards for workers in private industry	This allows states to adopt guidelines to manage the risks of NMs in the workplace
NIOSH	NMs	Developed interim guidance on the occupational safety and health implications and applications of NMs, including the use of effective control technologies, work practices, and personal protective equipment	

## The available methods for detection and characterization of NMs

Due to the unique chemical and physical properties of NMs (such as their size, structure, surface charge, and interactions in the environment), their detection, extraction, as well as analysis are considered a big challenge [4, 6]. Scientists focused on the environmental research of NMs, especially their fate, transport, and toxicological effects. They realized from the obtained results that their studies were hampered by a lack of adequate analytical techniques for the analysis at environmentally relevant concentrations in complex media. The conventional analytical techniques are not suitable for the physicochemical forms of NMs. Thus, it is essential to increase the number of research articles related to NMs with the development of techniques for extraction, cleanup, separation, and sample storage, increase sensitivity, and add specificity to analytical techniques [1, 2].

Analysis methods of NMs contaminants and environmental elements in environmental samples often include multiple technologies such as size separation mechanisms, particle counting systems, and morphological and/or chemical analysis technologies. Aerosol fractionation technologies are used to obtain NMs size fractions based on their mobility properties in an electrical field. Also, aerosol mass spectrometers are used in the chemical analysis of NMs suspended in gases and liquids through vaporization and analysis of the resulting ions [4]. NMs densities in gas suspension can be determined by Expansion Condensation Nucleus Counters with a detection limit of 3 nm through adiabatic expansion followed by optical measurement [133].

The extraction and size fractionation of NMs from liquid environmental samples can be done by size exclusion chromatography, ultrafiltration, and field flow fractionation. For further analysis of fractions of NMs, dynamic light scattering and mass spectrometry are used for size analysis and chemical characterization, respectively. Besides, for determining the size and shape of NMs (˂ 10 nm) we may use Scanning Electron Microscope (SEM) and/or Transmission Electron Microscope (TEM); and getting their morphological features in air and liquid media the Atomic Force Microscopy (AFM) is used. Moreover, for measuring the crystalline phase and determining the surface chemical composition and functionality of NMs, an X-ray diffractometer and X-ray photoelectron spectrometer are used respectively [4]. Finally, it is essential to develop and invent new methods and techniques for analyzing NMs contaminants in environmental samples.

## Technologies for controlling NMs contaminants in the environment

Groso et al. [134] adopted the application of the precautionary principle, especially with NMs. This is due to a lack of information that describes the health and environmental risk of engineered NPs or NMs, despite numerous discussions, reviews, and reports about nanotechnology [135]. Far from the management principles for controlling NMs in the environment, this review is focusing on the way for the treatment of these NMs to decrease their risk.

NMs are generally characterized by a high surface-to-volume ratio, which causes NPs to be extremely reactive. This high reactivity is one reason that makes NPs even much more harmful to the whole ecosystem, but at the same time facilitates methods of removing NPs contaminants. Metals and metal oxides are the most common NPs. They agglomerated with metallic quantum dots to form much larger particles, which can then be easily filtered out from the water in a conventional water treatment plant [135, 136]. NMs may be removed from groundwater, surface water, and drinking water by the processes of sand filtration, sedimentation, and flocculation [62]. It was found that there was a significant positive effect of an anionic sodium dodecyl sulfate and a nonionic nonylphenol ethoxylate, on ZnO NPs adsorption, aggregation, dissolution, and removal by the coagulation process [137]. Kirkegaard et al. [138] assessed tap water concentrations of the NPs (i.e., Ag, TiO_2_, and ZnO) by mass flow analyses of two wastewater treatment concepts: (1) advanced membrane treatment and (2) bank infiltration. They found that aggregation, sedimentation, coagulation, and biosorption were the main removal mechanisms of NPs in water. Thus, conventional biological treatment processes are effective barriers against NPs. They also noticed that the removal efficiency of advanced technologies [i.e., microfiltration (MF) and ultrafiltration (UF)] for ZnO NPs or Zn^+^ was low which could be mainly due to the hydrolysis or dissolution of ZnO NPs.

Removal of NMs can be done also by the adsorption process. For instance, metal NPs, in a colloidal solution of Mn, Cu, Zn, Ag + Ag_2_O, can be treated by adsorption with aquatic plants *Pistia stratiotes L.* and *Salvinia natans L.* [139], while complete removals of both ZnO NPs and CuO NPs in both single and binary suspensions from water were achieved by adsorption on activated carbon [140].

**Fig. 5:**
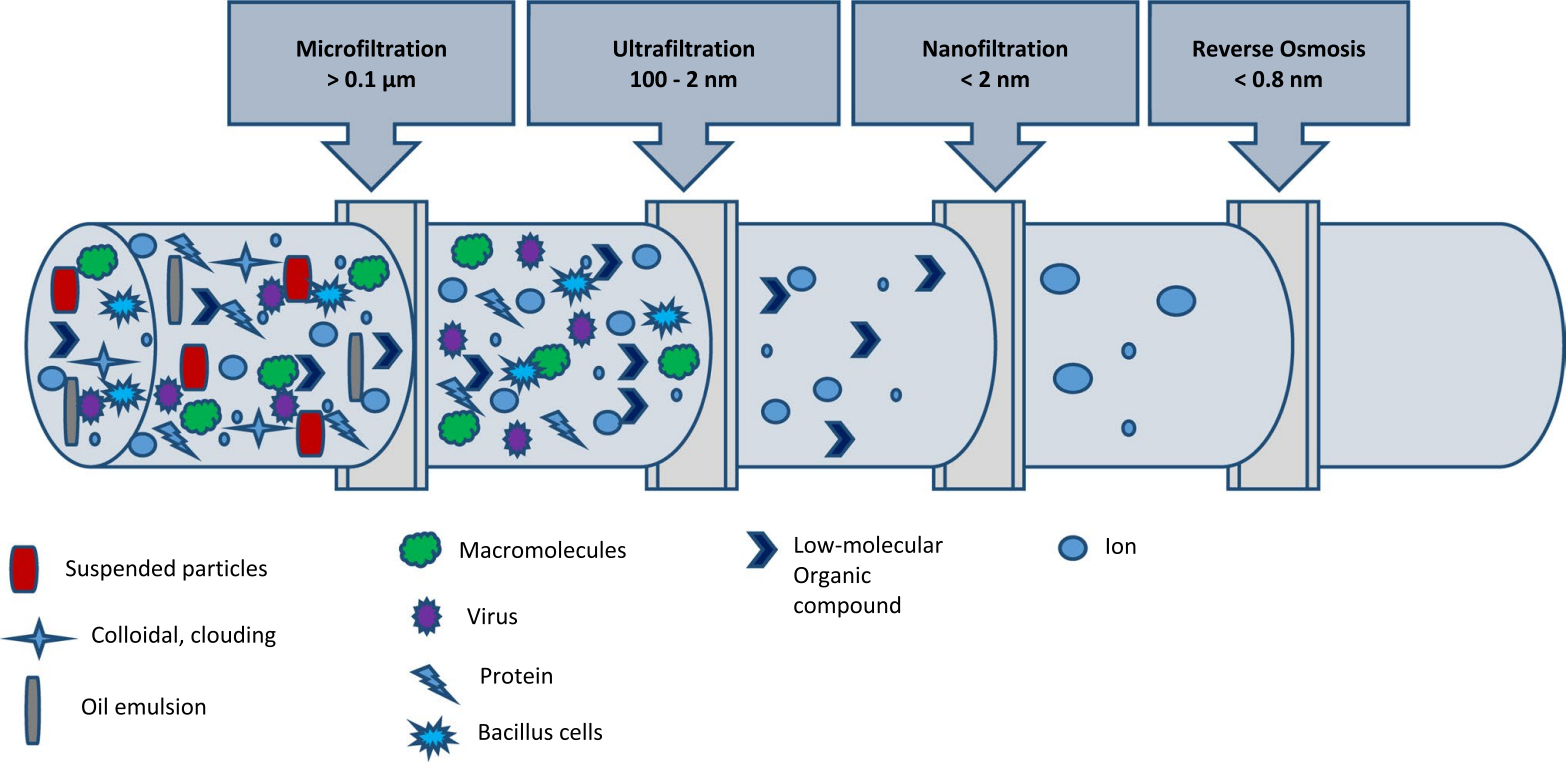
Removal possibilities of water pollutants using different filtration technologies

Adsorption and flocculation processes depend mainly on NPs surface charge, while membrane technology depends not only on the surface charge but also on the size of NPs. Membrane technologies get adapted in both aqueous and air media. Thus, it can play an important role in the removal of NMs emerging contaminants in the environment. Figure 5 shows the removal possibilities of water pollutants using different filtration technologies which are based on the size exclusion mechanism. According to the membrane pore diameter, UF is more effective for the removal of NPs. Nanofiltration (NF) and reverse osmosis (RO) are effective membrane filtration for NPs lower than 2 nm. However, the main drawback of such technology is the membrane fouling, which needs more efforts to solve this problem to increase the membrane lifetime and to decrease the treatment process cost.

Air filters and respirators are used to effectively remove NMs from the air [62]. The effective removal of NPs by fibrous filters has been previously reviewed [141].

## Conclusions and recommendations

Due to the involvement of nanotechnology in different sectors such as medicine, military, electronics, food, chemicals, energy, and a great variety of other scientific fields, it is so important to classify NMs and to unify the terms used for describing particle size. This will help scientists in the field of nanotechnology, health, and environmental sciences to easily determine the methods of detecting, controlling, and regulating NMs contaminants in the environment.

NMs are mainly characterized by a very small size (˂ 100 nm), which is responsible for increasing the surface area-to-volume ratio. This makes NMs more reactive than bulk forms of the same materials. Thus, predicting the chemical properties and potential toxicities of NPs is extremely difficult. Accordingly, it is necessary to develop detection and characterization methods for NMs to well define the properties of materials in nanoscale. This will help in detecting the mechanisms of NPs’ interaction with different environmental media and with living organisms. These mechanisms of interaction are essential for determining the fate, transport, and toxicity of environmental NMs contaminants.

The reactivity of NMs contaminants in the environment with different matrices leads to a recommendation for developing the extraction methods in terms of efficiency and simplicity. However, the cost of extraction methods should be taken into consideration as well.

There is a considerable large number of studies on human toxicity of NPs at a cellular level, which can hold for species other than humans. Thus, it is recommended not only to improve the existing test methods for toxicity but also to look for different species to have a full picture for the NPs’ impact in different environmental media and set specific standards and regulations for releasing.

In the case of emerging NMs, it is recommended to conduct risk assessment including the toxicity, exposure routes, environmental fate, transport, persistence, transformation, and recycling. Analysis of the life cycle will be useful for assessing the actual environmental impacts. Also, for manufacturing new NMs, it is essential to provide an effective strategy for recycling and recovery.

In the case of ENMs, adopting the application of the precautionary principle helps decrease the health and environmental risk of such materials. Also, following up the regulations concerning the use and handling of ENMs especially in the area of manufacturing will help a lot in decreasing their risk.

Due to the complexity of NMs, multiple analysis tools are recommended to have a complete picture of the analyte. This complexity leads us also to consider using the integration systems in the removal of NMs from water. For instance, flocculation and coagulation or adsorption should be integrated with a membrane technology to target the dissolved and suspended NMs at the same time. Moreover, membrane technology is recommended as a promising tool for the removal of NMs from the air.

Indeed, there are limited data on the fate, impact, toxicity, and risk of NMs; therefore, further research is essential to fill in this gap. Besides the setting of specific regulations and standards for NMs, it is necessary to minimize the release in the environment and to decrease the risk of handling and using these materials.

Developing an effective strategy for recycling and recovery of new NMs is crucial to mitigate potential negative impacts. An analysis of their life cycle can also assist in evaluating real environmental effects if NMs are released into the environment.

## Data Availability

All data produced or analyzed during this study are included in this published article.
